# Endotoxin Exposure during Sensitization to *Blomia tropicalis* Allergens Shifts TH2 Immunity Towards a TH17-Mediated Airway Neutrophilic Inflammation: Role of TLR4 and TLR2

**DOI:** 10.1371/journal.pone.0067115

**Published:** 2013-06-21

**Authors:** Renato Barboza, Niels Olsen Saraiva Câmara, Eliane Gomes, Anderson Sá-Nunes, Esther Florsheim, Luciana Mirotti, Alexis Labrada, Neuza Maria Alcântara-Neves, Momtchilo Russo

**Affiliations:** 1 Departamento de Imunologia, Instituto de Ciências Biomédicas, Universidade de São Paulo, São Paulo, Brazil; 2 Departmento de Alergenos, Centro Nacional de Biopreparados, La Habana, Cuba; 3 Instituto de Ciências da Saúde, Universidade Federal da Bahia, Bahia, Brazil; Centre de Recherche Public de la Santé (CRP-Santé), Luxembourg

## Abstract

Experimental evidence and epidemiological studies indicate that exposure to endotoxin lipopolysaccharide (eLPS) or other TLR agonists prevent asthma. We have previously shown in the OVA-model of asthma that eLPS administration during alum-based allergen sensitization blocked the development of lung TH2 immune responses via MyD88 pathway and IL-12/IFN-γ axis. In the present work we determined the effect of eLPS exposure during sensitization to a natural airborne allergen extract derived from the house dust mite *Blomia tropicalis* (Bt). Mice were subcutaneously sensitized with Bt allergens co-adsorbed onto alum with or without eLPS and challenged twice intranasally with Bt. Cellular and molecular parameters of allergic lung inflammation were evaluated 24 h after the last Bt challenge. Exposure to eLPS but not to ultrapure LPS (upLPS) preparation during sensitization to Bt allergens decreased the influx of eosinophils and increased the influx of neutrophils to the airways. Inhibition of airway eosinophilia was not observed in IFN-γdeficient mice while airway neutrophilia was not observed in IL-17RA-deficient mice as well in mice lacking MyD88, CD14, TLR4 and, surprisingly, TLR2 molecules. Notably, exposure to a synthetic TLR2 agonist (PamCSK4) also induced airway neutrophilia that was dependent on TLR2 and TLR4 molecules. In the OVA model, exposure to eLPS or PamCSK4 suppressed OVA-induced airway inflammation. Our results suggest that *B. tropicalis* allergens engage TLR4 that potentiates TLR2 signaling. This dual TLR activation during sensitization results in airway neutrophilic inflammation associated with increased frequency of lung TH17 cells. Our work highlight the complex interplay between bacterial products, house dust mite allergens and TLR signaling in the induction of different phenotypes of airway inflammation.

## Introduction

Asthma is a complex syndrome that affects more than 200 million people worldwide and is characterized by airway inflammation, obstruction and hyperreactivity [1]. Recent evidences indicate that asthma encompasses many different phenotypes ranging from classical TH2 allergic phenotype, characterized by airway eosinophilia, to a TH1 or TH17 dominated phenotypes, which are correlated with airway neutrophilic inflammation and production of IFN-γ and IL-17, respectively [2], [3]. TH1/TH17 phenotypes are frequently found in lungs of patients with severe asthma or asthma resistant to corticosteroids [3].

Murine models of asthma have been developed to study the inflammatory mechanisms of asthma [4], [5]. For this, mice are sensitized and challenged with different kinds of allergens, being ovalbumin (OVA) the most widely used antigen [6], [7]. However, the OVA model of allergic lung disease does not represent the real scenario to which asthmatics are normally exposed throughout their life. Thus, the murine model of asthma-like inflammation has been adapted to environmental airborne allergens such as those derived from house dust mites [8]. Many patients have positive skin prick tests to allergens derived from the mites *Blomia tropicalis* or *Dermatophagoides pteronyssinus* and sensitization to these mites is associated with asthma symptoms [9], [10]. Although the nature of allergen represents an essential component of asthma, other environmental factors such as endotoxin must also be considered in asthma development. Endotoxin is a high molecular complex derived from the cell wall of Gram-negative bacteria constituted mainly by lipopolysaccharides (LPS) that activate immune cells via Toll-like receptors (TLR) [11]. LPS is ubiquitous in the environment and it is often present in household dusts. Epidemiological studies claim that endotoxin exposure during childhood could protect against the development of asthma later in life while other studies indicate that endotoxin exposure is a risk factor for asthma [12], [13], [14], [15], [16]. Experimental data obtained from murine OVA model are in line with the dual role of LPS in asthma development [17]. It is now clear that the route, concentration, timing and duration of LPS exposure can determine whether LPS down- or up-regulates TH2-mediated allergic responses [17], [18].

Using the OVA model we have previously shown that absorption of LPS to alum, a TH2-prone adjuvant, during OVA priming inhibited TH2 responses in a dose-dependent manner without inducing a TH1-associated lung inflammation [19]. These results indicate that TLR agonists could potentially be used as adjuvants in formulations of vaccines to prevent or to treat allergy. In order to extend these observations, in this work we determined the effect of adsorption of TLR agonists to alum in a murine model of allergic asthma using *B. tropicalis* allergens. In addition, the OVA model was used for comparison. Strikingly, we found that *B. tropicalis* allergens, but not OVA, potentiate TLR2 signaling by concomitantly engaging TLR4. This dual role on TLRs activation results in airway neutrophilic inflammation that is mediated by TH17 cells.

## Materials and Methods

### Mice

Six- to eight-week-old C57BL/6, IFN-γ^−/−^, IL-17RA^−/−^, TLR2^−/−^, and TLR4^−/−^ mice were bred in our animal facilities at the Institute of Biomedical Sciences of University of São Paulo under standard pathogen-free conditions. TLR2^−/−^ and TLR4^−/−^ mice were kindly provided by Dr. Shizuko Akira (Osaka University, Japan), and IL-17RA^−/−^ mice were kindly provided by Dr. João Santana da Silva (School of Medicine of Ribeirão Preto, University of Sao Paulo, Brazil). All knockout mice used in this study were derived from C57BL/6 strain. Mice were housed under barrier conditions and provided with food and water *ad libitum*. All animals were fed with regular diet and all procedures were in accordance with national regulations on animal experimentation and welfare and were authorized by the Institutional Animal Care and Use Committee of the Institute of Biomedical Sciences – USP (CEUA#53fl46livro2).

### *B. tropicalis* allergens

*B. tropicalis* (Bt) house dust mites were collected from bed dust in Salvador, Brazil, cloned and cultured with a powdered fish food medium (Spirulina, Alcon Gold, São Paulo, Brazil), and dry yeast (Fermipan, São Paulo, Brazil), at 25°C and 75% humidity. Mites were harvested from the medium by flotation on a 5 M sodium chloride solution, followed by several washings with endotoxin-free distilled water using a 100 µm pore size polystyrene filter. The washings were carried out until no food residues could be seen under microscopy. Mites were suspended in 0.15 M phosphate-buffered saline, pH 7.4 (PBS) and lysed in a blender (Waring Commercial, Torrington, CT, USA). Lipids from the lysate were extracted with ether and discarded after five or six extractions. The protein content of the aqueous extract was determined by the method of Lowry [20] and stored at −70°C until use. The concentration of LPS on Bt extract was less than 0.0178 ng/µg as determined by Limulus amoebocyte lysate (LAL) QCL-1000 kit (BioWhittaker, Walkersville, MD, USA).

### Removal of LPS from ovalbumin

Chicken OVA (Sigma-Aldrich, St. Louis, MO, USA) was diluted in phosphate buffer saline (PBS) and depleted of the endotoxin activity by two to four cycles of Triton X-114 extractions. After the final extraction, OVA concentration was determined by BCA kit assay (Pierce Biotechnology Inc., Rockford, IL, USA) and adjusted to 2 mg/mL. The endotoxin level of purified OVA after LPS depletion was below the limit of detection (<0.01 ng/ml) as determined by Limulus amoebocyte lysate (LAL) QCL-1000 kit (BioWhittaker, Walkersville, MD, USA).

### Alum gel preparation

Alum (Al(OH)_3_) gel was prepared by precipitating 0.184 M ammonium aluminum sulphate dodecahydrate (AlH_4_-NO_8_S_2_ 12H_2_O) with an excess of 1 N NaOH, (roughly 2.5∶1 v/v). After precipitation, Al(OH)_3_ was suspended in Milli-Q water and washed five times at 1000 *g* for 15 min and its final concentration was calculated by determining the dry weight of 1 mL solution.

### Immunization and airway challenge

To induce allergic airway disease, mice were sensitized by subcutaneous injection (0.2 mL total volume) in the nape of the neck with Bt or OVA (5 µg per animal) in the presence of absence of TLR ligands (0.1–10 µg) co-adsorbed onto alum on days 0 and 7, followed by intranasal challenges with 10 µg of Bt or OVA in 50 µL of PBS on days 14 and 21. The first challenge recruits memory T cells to the lung that respond promptly upon the second challenge. Because of that, we have previously demonstrated that almost all allergic parameters (cell infiltration, antibodies and cytokine production) can be evaluated 24 h after second challenge. To test the effect of TLR agonists on established airway inflammation, we used LPS from *Escherichia coli* 0111:B4 (herein named eLPS - Sigma-Aldrich) and ultrapure LPS from *E. coli* K12 (herein named upLPS - InvivoGen, CA, USA), as TLR4 ligands; and Pam3CSK4 (InvivoGen) as TLR2 ligand.

### Bronchoalveolar lavage fluid and blood collection

Mice were deeply anaesthetized by an intraperitoneal injection of 4 mg/Kg of body weight of chloral hydrate (Labsynth, Sao Paulo, SP Brazil) and blood samples were collected by cardiac puncture for serum antibody level determinations. The trachea was cannulated, and lungs were washed twice with 0.5 mL of PBS. Total and differential cell counts of bronchoalveolar lavage (BAL) fluid were determined by haemocytometer and cytospin preparation stained with Instant-Prov (Newprov, PR, Brazil).

### Cytokine and antibody determinations

The levels of cytokines (IL-4, IL-5, IL-13, IL-17, VEGF and IFN-γ) in the BAL fluid were assayed by OptEIA™ ELISA sets according to the manufacturer's recommendation (BD Biosciences, PharMingen, San Diego, CA, USA), as previously described [21]. For IL-13 determinations, the antibodies pairs were purchased from R&D Systems (Minneapolis, MN, USA). Values were expressed as pg/mL deduced from standard curves of recombinant cytokines ran in parallel. The limits of detection were 5 pg/mL for IL-4 and 10 pg/mL for IFN-γ, IL-5, IL-13, IL-17, and VEGF. Measurement of total immunoglobulin E (IgE) was assayed by sandwich ELISA as previously described [30]. Briefly, plates were coated overnight at 4°C with 0.2 mg/mL of unlabelled rat anti-mouse IgE (Southern Biotechnology, Birmingham, AL, USA). Serum samples were added and bound IgE was revealed with biotin-labelled antibody followed by ExtrAvidin®-Peroxidase conjugate (Sigma-Aldrich). The levels of IgE were deduced from IgE standard curve ran in parallel. Bt-specific IgE antibodies were assayed by sandwich ELISA as previously described [8].

### Flow cytometry

Cells isolated from lung tissue after collagenase and DNAse digestion were surface stained with anti-Gr1 and anti-Siglec F antibodies (BD Biosciences). For intracellular staining of IL-17, IL-5 and IL-4 cells were cultured for 6 h at 37°C, 5% of CO_2_ in presence of Stop Golgi (BD Biosciences). After wash, cells were fixed, permeabilized (BD Cytofix/Cytoperm kit BD-PharMigen) and finally stained with anti-IL-17, IL-5 and anti-IL-4 antibodies (BD Biosciences). Cells were acquired by flow cytometry using a FACSCanto II (BD Biosciences) and data was analyzed by the FlowJo software, version 7.5.5 (Tree Star, Ashland, OR, USA).

### Histological analyses

After BAL collection, lungs were perfused via the right ventricle with 5 mL of PBS to remove residual blood, immersed in 10% phosphate-buffered formalin for 24 h, and then in 70% ethanol until embedded in paraffin. Lung sections of 5 µm were stained with periodic acid-Schiff (PAS)/haematoxylin for the evaluation of mucus production as described earlier [27]. A quantitative digital morphometric analysis was performed using the application program Metamorph 6.0 (Universal Images Corporation, Downingtown, PA, USA). The circumference area of bronchi and the PAS-stained area were electronically measured and the mucus index was determined by the following formula: (PAS-stained area/bronchial circumference area)/100.

### Protease enzyme activity assay

To study the putative role of proteases on the observed biological effects, *B. tropicalis* extract was inactivated by heat at 100°C for 15 minutes. The enzymatic activity analysis of extract was detected by hydrolysis of Z-F-R-MCA at λ = 460 nm/380 nm (emission/excitation wavelengths for MCA, respectively) using Hitachi F-2500 spectrofluorometer. Before the assay, proteases present in the whole *B. tropicalis* extract (inactivated or not) were activated by DTT1 (5 min a 37°C in PBS). After proteases activation, 5 mM Z-F-R-MCA were added to a 1-cm-pathlength cuvette containing 1 mL of the substrate solution. The cuvette was placed in the thermostatic cell compartment. The increase of fluorescence was continuously recorded for 5 min. The results are expressed in Arbitrary Units of Fluorescence (AUF).

### Macrophage nitric oxide production

TLR4^−/−^ mice were injected with 3% aged thioglycollate broth (Difco Laboratories, Detroit, MI, USA) and after 4 days, the peritoneal cells were collected by lavage with 3 mL of cold medium (RPMI-1640, GIBCO-Invitrogen, Grand Island, NY, USA). A suspension containing 1.5×10^6^ cells/mL was distributed in 96-well flat bottom plates at 100 µL aliquots/well and after 2 h, three washings with warm medium removed non-adherent cells. Cells were then incubated with complete medium (RPMI-1640 supplemented with 10% fetal bovine serum, 100 U/mL penicillin, 100 µg/mL streptomycin, 2 mM L-glutamine, and 0.05 mM 2-mercaptoethanol) plus IFN-γ (10 ng/mL) alone or in presence of increasing concentrations of eLPS or upLPS. Cell-free supernatant was removed after 48 h culture and nitric oxide (NO) production was estimated by determination of nitrite (NO_2_^−^), the stable product of NO oxidation, by standard Griess reaction [22].

### Statistical analysis

Results were compared by analysis of variance (ANOVA) followed by the Bonferroni post hoc test using GraphPad Prism (GraphPad Software Inc., version 5.00, CA, USA). A *p* value of 0.05 or less was considered statistically significant. Data are expressed as mean ± standard error of the mean (SEM).

## Results

### Allergen-induced airway neutrophilic inflammation is driven by eLPS exposure during sensitization to *B. tropicalis* allergens

**Figure 1:**
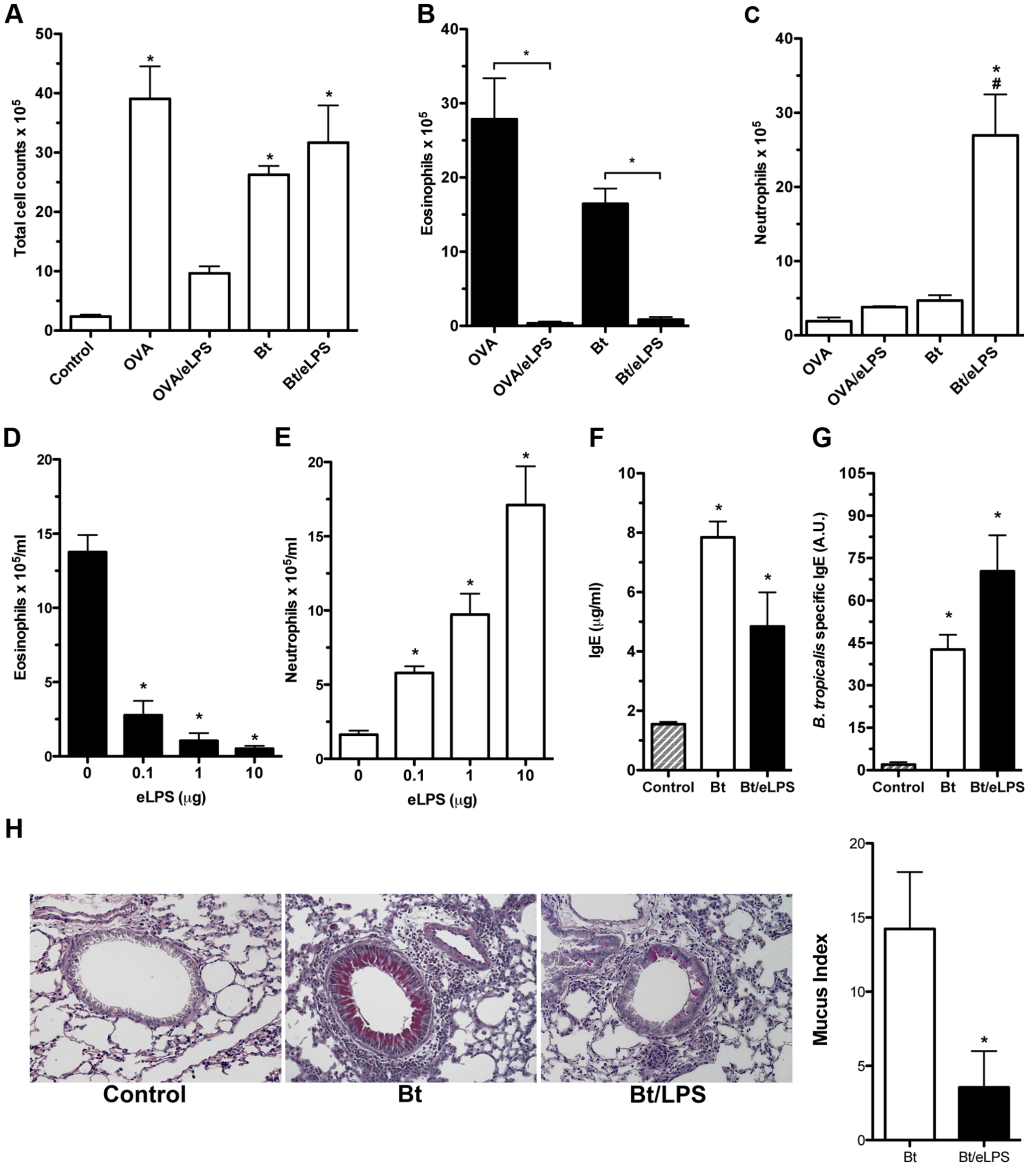
*B.* *tropicalis* allergens co-adsorbed with eLPS to alum induces a non-classical allergic airway disease phenotype. C57BL/6 (WT) mice were sensitized twice with Bt or Bt plus eLPS co-adsorbed to alum and challenged twice with Bt. Control group consisted of non-manipulated animals. The experiments were performed 24 h after the last Bt challenge. Same protocol was performed using OVA as allergen. Total bronchoalveolar lavage (BAL) leukocyte counts (**A**); Eosinophils (**B**) and neutrophils (**C**) counts in the BAL fluid; Effects of eLPS dose response on eosinophils (**D**) and neutrophils (**E**) counts in the BAL fluid, total (**F**) and Bt-specific (**G**) IgE in serum; Representative lung sections staining with periodic acid-Schiff (PAS) showing normal lung histology in control group (*left*), Bt group (*middle*), Bt/LPS group (*right*), and Mucus index (**H**). Results are expressed as mean ±SEM for groups of five mice and are representative of two experiments. *Significant difference (*p*<0.05) when compared with the control group or as indicated.

We have previously shown that sensitization to OVA in the presence of eLPS inhibited the development of TH2-mediated eosinophilic airway inflammation without shifting lung immunity towards TH1 pattern [19]. Because OVA is a typical food allergen we thought to evaluate whether eLPS could also suppress the development of allergic lung disease using natural respiratory allergens present in Bt, using the Bt model that we have developed [8]. By analyzing the degree of lung inflammation we found that sensitization with alum as adjuvant and challenge with OVA or Bt allergens increased the total number of cells in BAL fluid when compared with non-sensitized control group (**Fig. 1A**). In addition, the cellularity of BAL fluid from both OVA and Bt groups was similar, with clear predominance of eosinophils (**Fig. 1B and 1C**). However, when eLPS was added during sensitization, there was a clear distinction in the cellular compositions of BAL cells of OVA group when compared with Bt group (**Fig. 1A**). A significant inhibition of total BAL leukocyte influx and eosinophils was observed in the OVA/eLPS group when compared with allergic OVA group (**Fig. 1A**). In contrast, addition of eLPS during Bt sensitization did not affect the total BAL leukocyte influx when compared with allergic Bt group (**Fig.1A**). Nevertheless, the cellular composition of the BAL fluid was markedly different when comparing the Bt/eLPS with Bt group (**Fig. 1A**). In Bt/eLPS group, eosinophils were virtually absent while a significant increase in the number of neutrophils was observed (**Fig. 1B and 1C**). Next, we found that the inhibition of eosinophilia with concomitant increase in airway neutrophilia induced by eLPS occurred in a dose-dependent manner (**Fig. 1D and 1E**). These results show that, depending on the allergen employed, eLPS either inhibits lung inflammation (in the case of OVA) or shifts airway inflammation from eosinophilic to a neutrophilic pattern (in the case of Bt allergens).

We also evaluated whether IgE antibody production was affected by eLPS. In **Fig. 1F and 1G** we show that the total and specific IgE production were not significantly altered in the Bt/eLPS group when compared with Bt group. Finally, we evaluated the effects of eLPS on mucus production. As shown in **Figure 1H**, compared to Bt group, addition of eLPS to Bt preparations significantly inhibited mucus production. Our results indicate that eLPS, decrease mucus production but does not affect IgE production.

Because house dust mite extract contains serine and/or cysteine proteases that are known to have pro-allergic effect [23], [24], [25], we performed experiments with heat-inactivated Bt allergens that are devoid of protease activity (**Figure S1A**). No significant differences were observed in experiments performed with protease heat-inactivated Bt allergens or crude Bt allergens (**Figure S1B**).

### eLPS co-adsorbed with *B. tropicalis* allergens decreases type 2 cytokines and increases airway IL-17 and IFN-γ production

**Figure 2:**
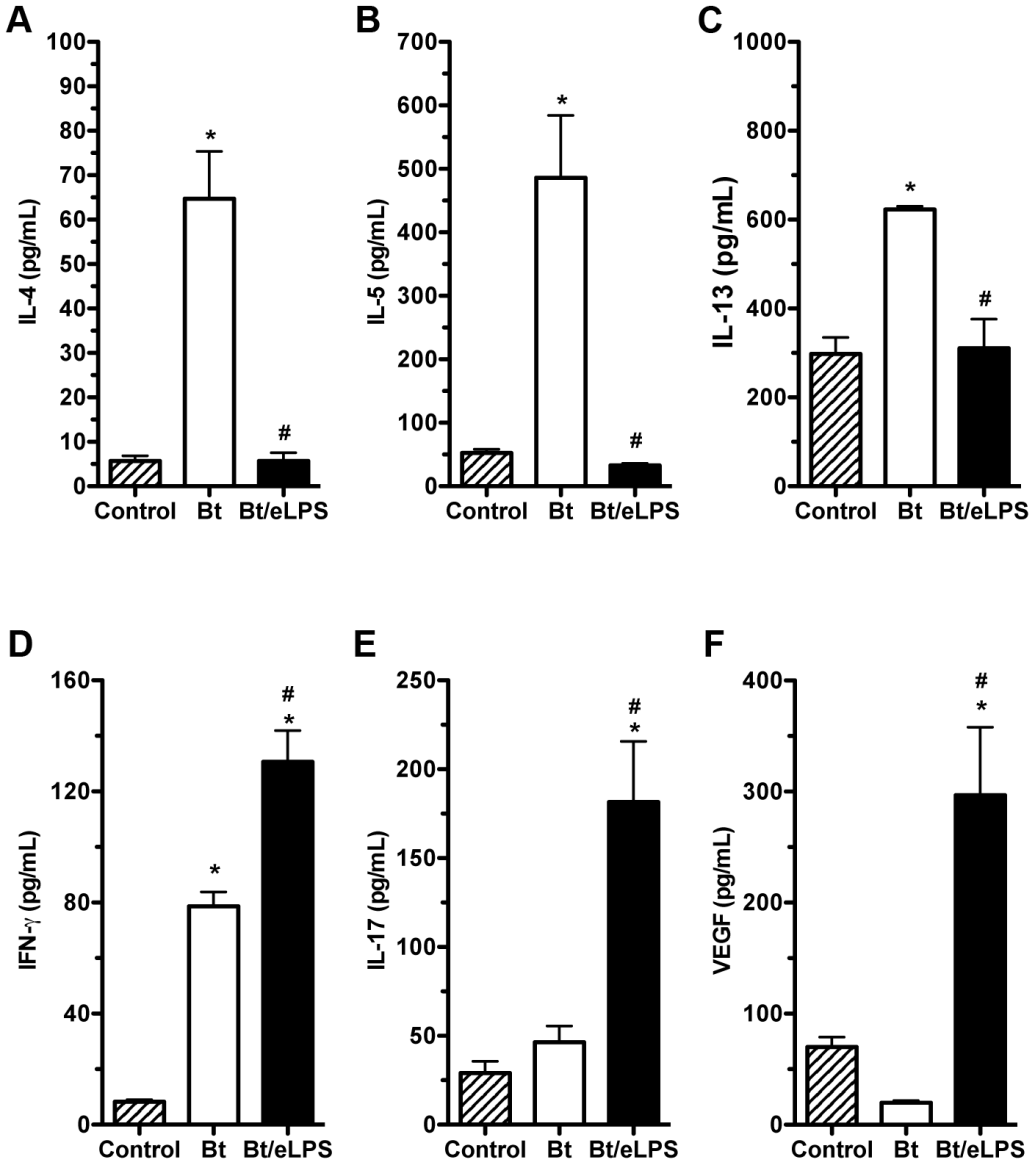
Airway inflammation induced by *B.* *tropicalis* allergens plus eLPS presented a TH1/TH17 pattern of cytokine production. C57BL/6 (WT) mice were sensitized twice with Bt or Bt containing eLPS co-adsorbed to alum and challenged twice with Bt. Experiments were performed 24 h after the last Bt challenge. Control group consisted of non-manipulated animals. Cytokine IL-4 (**A**), IL-5 (**B**), IL-13 (**C**), IFN-γ (**D**), IL-17 (**E**), and VEGF (**F**) production BAL fluid were measure by ELISA. *Significant difference (*p*<0.05) when compared with control group. ^#^Significant difference (*p*<0.05) when compared with Bt group.

To further characterize the neutrophilic airway inflammation induced in eLPS/Bt group, we compared the cytokine levels present in BAL fluid of these animals to those in the absence of eLPS. We also measured the production of vascular endothelial growth factor (VEGF) since it has been shown that it plays a critical role in TH2 inflammation [26]. As shown in **Figure 2**, animals that received eLPS showed a significant reduction in TH2-associated cytokines (IL-4, IL-5 and IL-13) with a concomitant increase in the levels of IFN-γ, IL-17 and VEGF, when compared with Bt group (**Fig. 2A, 2B and 2C**). These results indicate that sensitization with Bt plus alum in the presence of eLPS reduces TH2 cytokines and increases the production of TH1/TH17-related cytokines and VEGF.

### eLPS co-adsorbed to *B. tropicalis* allergens inhibit eosinophil influx via IFN-γ and increases neutrophil influx via IL-17

**Figure 3:**
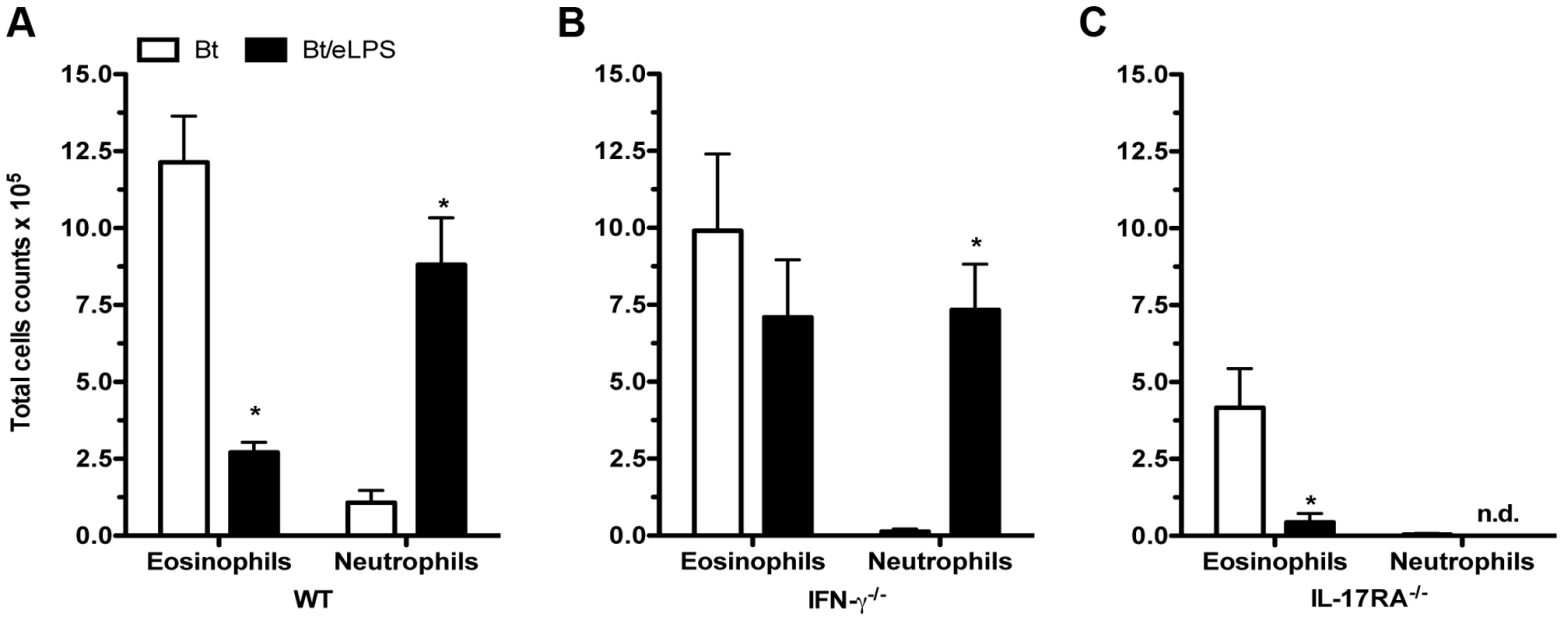
Neutrophilic airway inflammation induced by *B.* *tropicalis* allergens plus eLPS is dependent on IL-17, while eosinophilia inhibition depends on IFN-γ production. C57BL/6 (WT), IFN-γ^−/−^, or IL-17RA^−/−^ mice were sensitized twice with Bt or Bt containing eLPS co-adsorbed to alum and challenged twice with Bt. Control group consisted of non-manipulated animals. The experiments were performed 24 h after the last Bt challenge. WT (A), IFN-γ^−/−^ (B), and IL-17RA^−/−^ (C) eosinophils and neutrophils in BAL fluid. Results are expressed as mean ±SEM for groups of five mice and are representative of two experiments. (n.d.: not detected); *Significant difference (*p*<0.05) when compared with Bt group.

Because it has been shown that airway inflammation dominated by neutrophils can be associated with TH1 or TH17 cells [3], we next evaluate the effect of eLPS in IFN-γ-or IL-17RA-deficient mice. We found that wild-type (WT), IFN-γ- or IL-17RA-deficient mice sensitized with Bt allergens developed an airway inflammation dominated by eosinophils as revealed by differential cell counts in BAL (**Fig. 3**). However, the number of eosinophils was lower in IL-17 RA-deficient when compared with WT or IFN-γ-deficient mice indicating that IL-17 is involved in the development of full TH2-mediated eosinophilic inflammation [27]. The results obtained with eLPS differed markedly in IFN-γ^−/−^ mice and IL-17RA^−/−^ when compared with WT mice. In the latter, eLPS suppressed eosinophilia and induced airway neutrophilia (**Fig. 3A**). In contrast, in the presence of eLPS, IFN-γ-deficient mice increased neutrophil content but did not suppress eosinophil influx (**Fig. 3B**). We found that the levels of IL-5 production in both Bt and Bt/eLPS groups from IFN-γ^−/−^ mice were significantly higher than that of control group while IL-17 production was higher in Bt/eLPS group compared with Bt or control group (data not shown). Thus, the levels of IL-5 and IL-17 correlated respectively with eosinophil and neutrophil influx observed in IFN-γ^−/−^ mice. In IL-17RA^−/−^ mice addition of eLPS suppressed eosinophilia but did not induce airway neutrophilia (**Fig. 3C**). Surprisingly, in IL-17RA^−/−^ mice, we could not detect significant differences in the levels of IL-5 or IFN-γ among the groups (data not shown). Altogether, these results suggest that IFN-γ production appears to be required for the inhibition of airway eosinophilia whereas IL-17 signaling is essential for the induction of airway neutrophilia.

### Airway neutrophilia is dependent on MyD88 adaptor molecule and on CD14, TLR4 and TLR2 molecules

**Figure 4:**
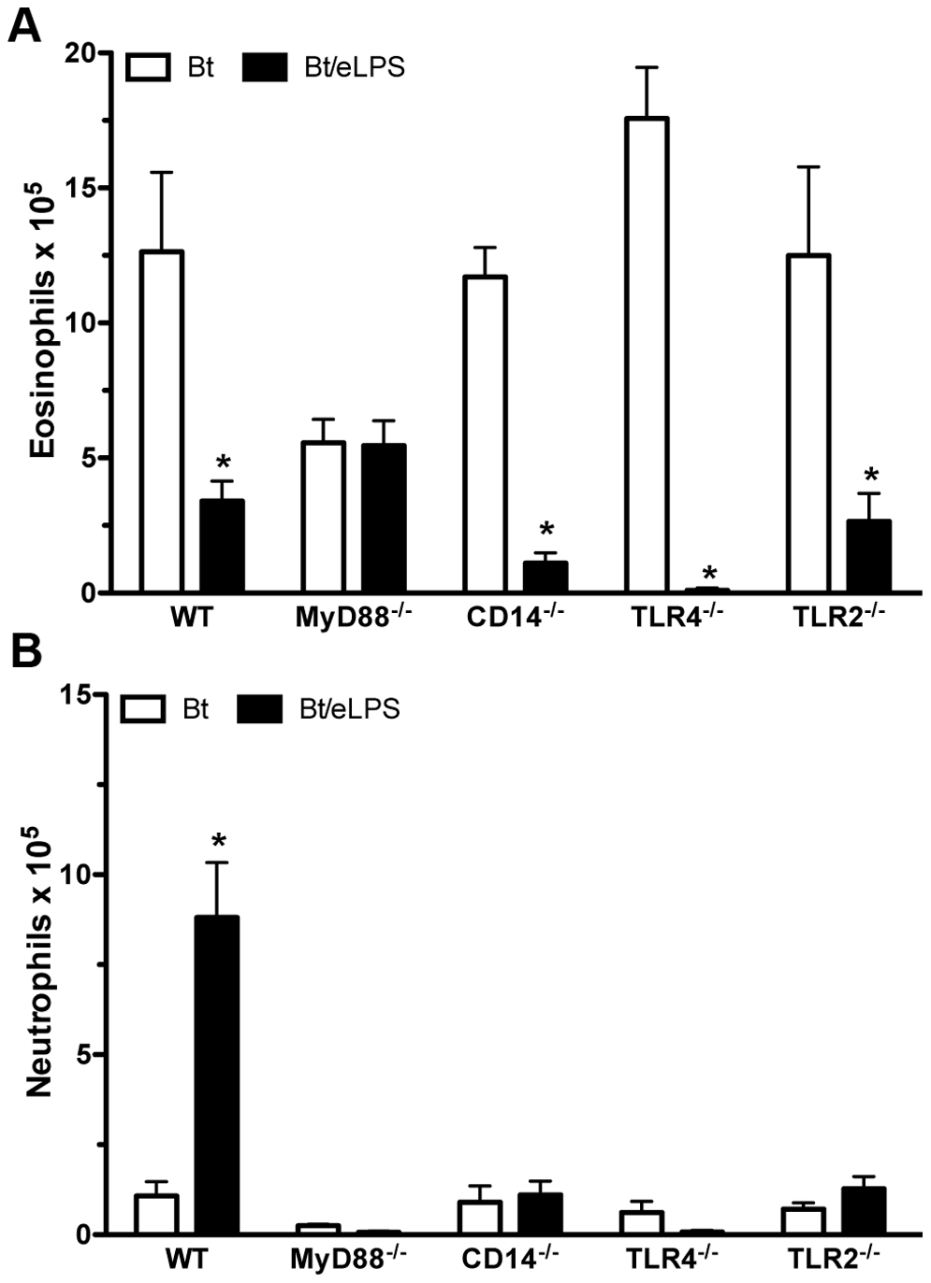
Development of airway inflammation in the presence of eLPS involves other components than just LPS. C57BL/6 (WT), MyD88^−/−^, CD14^−/−^, TLR4^−/−^, or TLR2^−/−^ mice were sensitized twice with Bt or Bt containing eLPS co-adsorbed to alum and challenged twice with Bt. Control group consisted of non-manipulated animals. The experiments were performed 24 h after the last Bt challenge. Eosinophils (**A**) and neutrophils (**B**) in BAL fluid. Results are expressed as mean ±SEM for groups of five mice and are representative of two experiments. *Significant difference (*p*<0.05) when compared with Bt group.

Since exposure to eLPS during sensitization modified Bt-induced airway inflammation, we next dissected the molecular pathways responsible for this effect. We first evaluated the role of MyD88, a key adaptor molecule involved in TLR signaling. We found that exposure to eLPS did not modify TH2 sensitization in mice lacking MyD88 (MyD88^−/−^) as airway responses to Bt challenges resulted in eosinophilic and not neutrophil dominated airway inflammation in these mice, which was similar to mice sensitized to Bt in the absence of eLPS (**Fig. 4A and 4B**). Therefore, the MyD88 adaptor molecule is essential to shift Bt-induced eosinophilia to airway neutrophilia in animals exposed to eLPS during sensitization. Endotoxin engages the LPS binding protein and binds to the cell surface CD14 that acts as co-receptor of the TLR4/MD-2 LPS receptor complex [28]. So, we also performed experiments in gene-targeted mice deficient for CD14, TLR4 and also TLR2. Bt sensitization and challenge of CD14^−/−^, TLR4^−/−^, or TLR2^−/−^ mice in absence of eLPS induced a classical TH2-mediated airway eosinophilia in all strains (**Fig. 4**), indicating that these molecules are dispensable for the induction of TH2 immunity. Addition of eLPS during Bt sensitization resulted in inhibition of airway eosinophilia in WT and also in CD14^−/−^, TLR4^−/−^ and TLR2^−/−^ mice (**Fig. 4A**), suggesting that these molecules are dispensable for the inhibition of airway eosinophilia induced by eLPS. Conversely, when we determined the influx of neutrophils in animals exposed to eLPS we found airway neutrophils were only present in WT mice, but not in mice deficient in MyD88, CD14, TLR4 and TLR2 molecules (**Fig. 4B**). Altogether, these results indicate that the mechanism(s) operating in the inhibition of airway eosinophilia are MyD88-dependent, but independent of CD14, TLR4 and TLR2. On the other hand, airway neutrophilia is also MyD88-dependent but, in addition, require the expression of at least CD14, TLR4 or TLR2. Therefore, it appears that eLPS utilizes different pattern recognition receptors that act in concert to drive the airway responses toward neutrophilic inflammation. One possibility is that eLPS could engage CD14, TLR2 and TLR4 simultaneously. In fact, it has been previously reported that many commercial preparations of eLPS are able to activate both TLR4 and TLR2 [**29]**, [30].

### Exposure to upLPS during sensitization to *B. tropicalis* allergens inhibits airway eosinophilia but does not induce airway neutrophilia

**Figure 5:**
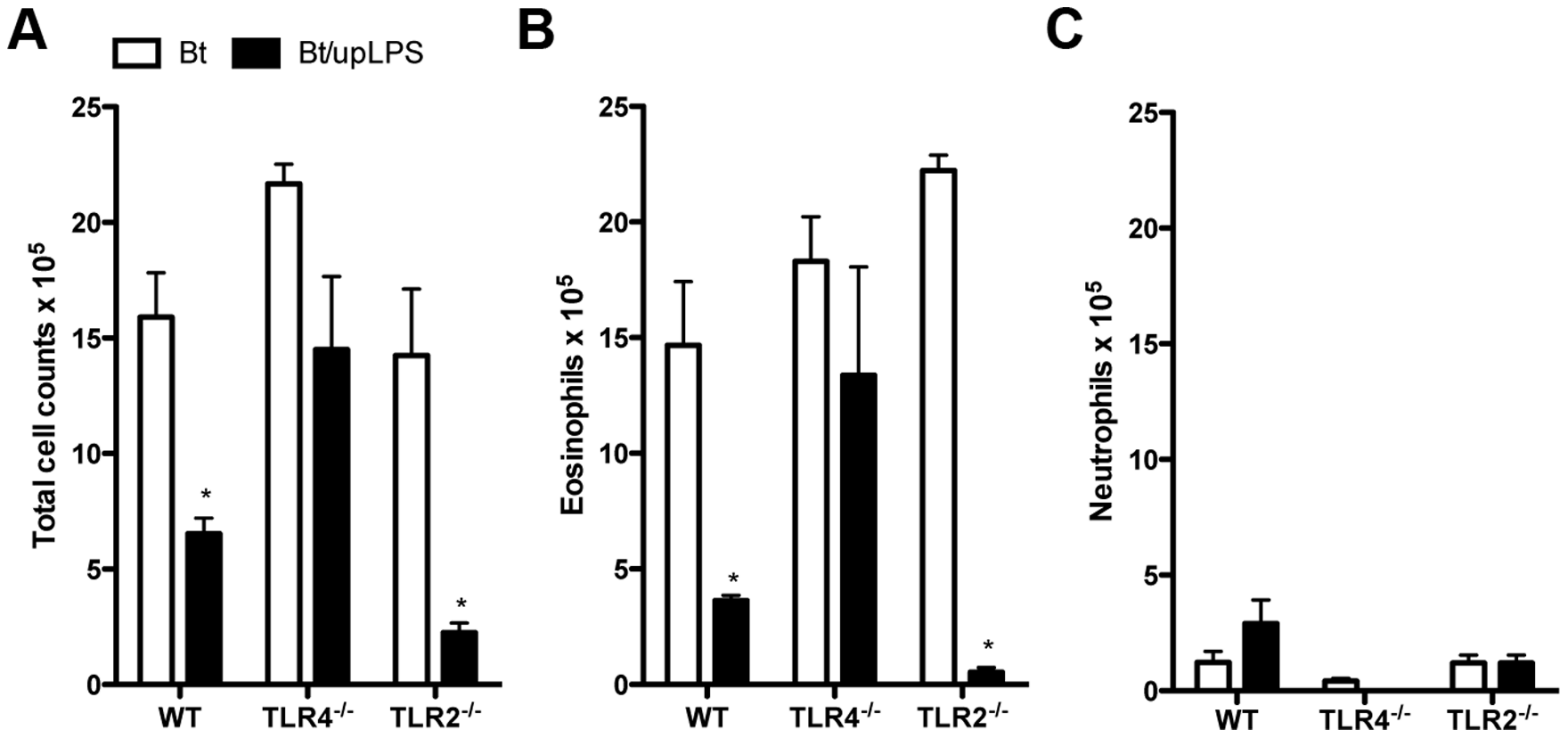
TLR4 activation by (upLPS) suppresses airway eosinophilia induced by *B.* *tropicalis* allergens. C57BL/6 (WT), TLR4^−/−^, or TLR2^−/−^ mice were sensitized twice with Bt or Bt containing upLPS co-adsorbed to alum and challenged twice with Bt. Control group consisted of non-manipulated animals. The experiments were performed 24 h after the last Bt challenge. Total bronchoalveolar lavage (BAL) leukocyte counts (**A**), eosinophil counts (**B**) and neutrophil counts (**C**) in BAL fluid. Results are expressed as mean ±SEM for groups of five mice and are representative of two experiments. *Significant difference (*p*<0.05) when compared with Bt group.

In order to assure specificity to TLR4-driven responses, we next used an ultrapure lipopolysaccharide preparation (upLPS) to evaluate its effects on Bt sensitization in WT, TLR2^−/−^ or TLR4^−/−^ mice. Results in **Figure 5** show that exposure to upLPS during allergen sensitization inhibited Bt-induced total leukocyte influx to BAL and BAL eosinophilia in WT and TLR2-deficient but not TLR4-deficient mice (**Fig. 5A and 5B**) indicating that upLPS preparation signals via TLR4 but not TLR2. Notably, exposure to upLPS did not result in a shift towards a neutrophilic airway inflammation as observed to eLPS (**Fig. 5C**). These results indicate that signaling through TLR4 molecule during Bt/Alum sensitization results in inhibition of effector T cells responsible for allergic eosinophilic inflammation, but it is insufficient to induce effector T cells that drive airway inflammation towards a neutrophilic pattern.

To confirm that eLPS preparation was signaling through a TLR4-independent pathway, we compared the ability of eLPS or upLPS in inducing nitric oxide (NO) production *in vitro* in IFN-γ primed macrophages cultures from TLR4^−/−^ mice. Our results showed that eLPS but not upLPS increased NO production in a concentration-dependent manner (**Figure S2**). These results clearly indicated that upLPS only signals through TLR4 molecule while eLPS signals by a TLR4-dependend and independent manner.

### Exposure to Pam3CSK4, a synthetic TLR2 agonist during sensitization to *B. tropicalis* allergens inhibits airway eosinophilia and induces airway neutrophilia

**Figure 6:**
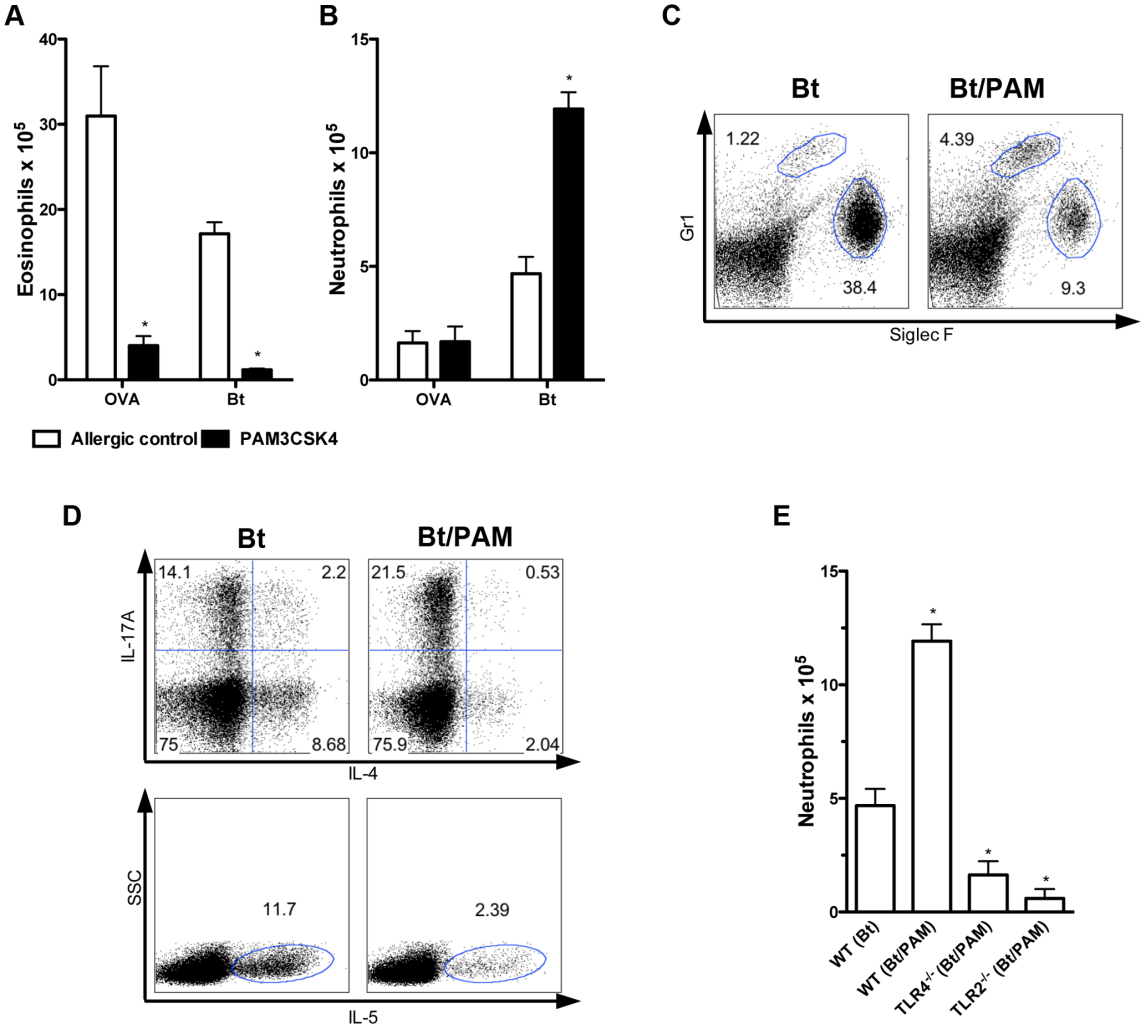
Endotoxin contamination with a TLR2 agonist is responsible by airway neutrophilia observed in mice sensitized with *B.* *tropicalis* allergens plus eLPS. C57BL/6 (WT), TLR2^−/−^ or TLR4^−/−^ mice were sensitized twice with Bt or Bt containing PAM3CSK4 (PAM) co-adsorbed to alum and challenged twice with Bt. Control group consisted of non-manipulated animals. The experiments were performed 24 h after the last Bt challenge. Same protocol was conducted using OVA as allergen instead Bt. Eosinophil (A) and neutrophil (B) counts in BAL fluid. Representative dot plot analysis of eosinophils (Gr-1^Low^/Siglet-F^High^) and neutrophils (Gr-1^High^/Siglec-F^low^) of mice sensitized in the presence of PAM3CSK4 (c). Intracellular staining for IL-4, IL-5 and IL-17 cytokine production (D), and BAL neutrophils counts (E). Results are expressed as mean ±SEM for groups of five mice and are representative of two experiments. *Significant difference (p<0.05) when compared with Bt group.

Since airway neutrophilia was only observed in mice exposed to eLPS, but not to upLPS during sensitization to Bt allergens, we postulated that in addition to TLR4, TLR2 could also play a role on this phenomenon. In order to test this possibility, we co-adsorbed Pam3CSK4, a synthetic tripalmitoylated lipopeptide that mimics the acylated amino terminus of bacterial lipoproteins and signals via TLR2 receptor [21], [31], [32] to alum containing Bt allergens. For comparison, we also tested the effect of Pam3CSK4 using OVA as allergen. **Figure 6A** shows that Pam3CSK4 inhibited the influx of eosinophils in both, OVA and Bt models; however in sharp contrast to OVA model, exposure to Pam3CSK4 during allergen sensitization resulted in the development of airway neutrophilia after Bt challenge (**Fig. 6A**). Flow cytometric analysis performed to distinguish eosinophils (Gr-1^Low^/Siglet-F^High^) from neutrophils (Gr-1^High^/Siglec-F^Low^) confirmed that sensitization in the presence of Pam3CSK4 resulted in a shift towards neutrophilic inflammation (**Fig. 6B**). We next determined the intracellular production of IL-4, IL-5 or IL-17A by CD4^+^ T cells in the Bt model. **Figure 6C** shows that, in the Bt group, CD4^+^ T cells produced IL-17, IL-4 and IL-5 whereas in the Bt/Pam3CSK4 group, the production of type 2 cytokines (IL-4 and IL-5) decreased while IL-17A increased. These results suggested that sensitization to Bt allergens in the presence of a synthetic TLR2 agonist shifts TH2 towards TH17 immunity. To assure ligand specificity, we also determined whether PamCSK4 would induce airway neutrophilia in TLR2^−/−^ or TLR4^−/−^ mice. As expected, airway neutrophilia was not observed in TLR2-deficient mice (**Fig. 6E**). However, TLR4-deficient mice exposed to PamCSK4 during sensitization also did not develop airway neutrophilia. We conclude that both TLR2 and TLR4 are required for Bt-induced airway neutrophilia. Since PamCSK4 is a pure TLR2 agonist, the TLR4 engagement probably derives from the interaction with Bt allergens. It follows, that during sensitization, Bt allergens engage TLR4 that in the presence of TLR2 agonist potentiates TLR2 signaling that result in the development of TH17 immunity.

## Discussion

Murine asthma-like models reproduce the immunological events underlying TH2 immunity. To achieve experimental TH2 immunity allergens are commonly adsorbed onto alum. Although the use alum-based models does not represent a real scenario for allergic sensitization in humans, they provide a rapid and reproducible system for studies on the modulation of allergic response as alum is a prototypic type 2 adjuvant [6]. Indeed, alum-based sensitizations with different kinds of allergens such as OVA (the most widely used allergen), house dust mite and plant extracts induce TH2-mediated responses upon allergen challenges [6], [8], [33], [34]. Here we confirmed that animals sensitized with Bt allergens adsorbed onto alum develop allergic lung disease similar to the OVA model. Using the OVA model we have previously shown that exposure to eLPS during OVA/alum sensitization inhibited dose-dependently TH2 responses without shifting the responses towards a TH1 pattern [35]. These results indicate that TLR agonists could be potentially used in formulations of vaccines against allergy. Here we confirmed that exposure to eLPS during OVA sensitization inhibits the development of TH2 immunity. However, quite unexpectedly, we found that exposure to eLPS during sensitization to Bt allergens did not prevent the development of airway inflammation, as was the case of OVA allergen. Instead, we found that mice sensitized to Bt allergens in the presence of eLPS developed airway inflammation dominated by neutrophils upon Bt challenges. The shift from eosinophil to neutrophil dominated airway inflammation induced by sensitization in the presence of eLPS was dose-dependent. Previous report by Carvalho et al. (2004) comparing airway inflammation induced by allergens from *B. tropicalis* or *D. pteronyssinus* (Dp) showed that Bt-challenged mice developed a higher neutrophilic response when compared with Dp-challenged mice [36]. However, the authors did not determine the level of endotoxin present in their extracts. As shown here, endotoxin contributes in a dose-dependent manner for the shift of eosinophilic allergic inflammation towards airway inflammation dominated by neutrophils. Indeed, because we removed LPS from our Bt extract we only found significant infiltration of neutrophils when we sensitized the animals in the presence of eLPS indicating the Bt allergens *per se* do not induce airway neutrophilia.

It is known that TH1- or TH17-mediated immune response can be accompanied by neutrophilic infiltration [3], [37], [38]. We found that the shift from eosinophilic to neutrophilic airway inflammation was paralleled by increased levels of IFN-γ and IL-17 and decreased levels of IL-5 in BAL. Experiments in IFN-γ or IL-17RA-deficient mice revealed that IFN-γ was responsible for the inhibition of airway eosinophilia whereas IL-17 was required for the induction of airway neutrophilia. It was previously shown the IFN-γ suppresses eosinophilic airway inflammation [39], [40] whereas IL-17 has dual role in experimental asthma [41], [42]. In our study, IL-17RA-deficient mice developed the lowest TH2 immunity when compared with WT and IFN-γ-deficient mice indicating that this strain has intrinsic refractorines to develop full lung allergy. In line with this observation, Besnard *at al.* showed that the production of IL-22 and IL-17 by TH17 cell are critical for the establishment of TH2 allergic asthma [42]. We found that eLPS did not suppress airway eosinophilia in MyD88- or IFN-γ ˜deficient mice while suppression was observed in mice deficient in CD14, TLR4 or TLR2 molecules. In contrast, induction of airway neutrophilia was not observed in mice deficient in CD14, or TLR4 or TLR2 indicating the key role of these molecules in the induction of airway neutrophilia. These results indicated that eLPS is exerting its activity by signaling also via TLR2. Indeed, our eLPS preparation could induce NO production in TLR4-deficient macrophages indicating that eLPS also signals via TLR4-independent pathway. To further dissect the role of TLR2 and TLR4 signaling we performed experiments with pure preparations of TLR4 or TLR2 agonists. Thus, we found that upLPS did not affect allergic sensitization in TLR4-deficient mice. Notably, in WT or TLR2-deficient mice exposure to upLPS preparation suppressed allergic inflammation but did not provoke airway neutrophilic inflammation. Therefore, during alum-based TH2 sensitization to Bt allergens in the presence of upLPS, that only signals via TLR4 molecule, is essentially inhibitory of TH2 responses. In contrast, strict TLR2 signaling by a synthetic TLR2 agonist (Pam3CSK4) during sensitization resulted in Bt-induced airway inflammation dominated by neutrophils in WT mice. However, TLR2 signaling is not sufficient to induce neutrophilia because in TLR4-deficient mice, exposure to Pam3CSK4 did not induce airway neutrophilic inflammation. We concluded that both, TLR2 and TLR4 molecules are required for the induction airway neutrophilic inflammation. Because in the OVA model, exposure of WT mice to eLPS or Pam3CSK4 did not result in neutrophilic inflammation, we reasoned that the difference between OVA and Bt allergens might be related to the capacity of Bt allergens in engaging TLR4 molecules. In consonance with this hypothesis, it was previously shown that Der p 2, a major allergen from the *D. pteronyssinus* mite has structural mimicry with MD-2, the LPS-binding component of the TLR4 signaling complex [43]. This characteristic could be applied to allergens of *B. tropicalis* due the similarities between the allergens from both species, as described for Blo t 5, a major allergen of *B. tropicalis*, and Der p 5 [44], [45], [46] Indeed, several airborne allergens are lipid-binding proteins and might activate TLR4 pathway. Previous work of Dziarski et al. showed that MD-2 associates physically with TLR2 and enhanced TLR2-mediated responses [47]. With regard to Bt extract used, analysis of protein fractions by SDS-polyacrylamide gel electrophoresis showed a band fraction of about 14.5 KDa that encompass many allergens such as Blo t 2, Blo t 5 and Blo t 12 that potentially could engage TLR4 (data not shown). Therefore, to explain our results we envisage the following scenario: Bt allergens engage TLR4 molecules that in turn potentiate TLR2 signaling by eLPS or Pam3CSK4. This dual activation of TLR4 and TLR2 leads to TH17 immunity and neutrophilic inflammation via MyD88 adaptor molecule. It follows that despite of the strong TH2 effect of alum, Bt allergens plus exogenous TLR2 agonist result in the dual activation of TLR2/TLR4 that dampened the TH2 driving effect of alum changing the immunity towards a TH17 pattern. It should be noted that endotoxin that is often present in household dusts, can potentially activate TLR4 by the LPS molecule and TLR2 by other cell wall components present in gram-negative bacteria such as lipid associated peptides or peptidoglycans [30], [48]. The engagement of TLR2 and TLR4 molecules during house dust mite sensitization leads to an asthma phenotype dominated by neutrophils and TH17 cells, a clinical feature that represent a more severe asthma [49]. In conclusion, our work highlights the complex interplay between bacterial products, house dust mite allergens and TLR signaling in the induction of different phenotypes of airway inflammation.

## Supporting Information

Figure S1 **Endotoxin LPS (eLPS) and Ultra-pure LPS (upLPS) have distinct effects on** ***in vitro*** **activation of TLR4^−/−^ macrophages.** Thioglycollate-elicited peritoneal macrophages from C57BL/6 (WT) or TLR4^−/−^ mice were incubated with medium only or stimulated *in vitro* with IFN-γ alone (10 ng/mL) or in the presence of different concentrations of eLPS or upLPS. NO_2_^−^ production was evaluated after 48 h culture by Griess reaction. *Significant difference (*p*<0.05) when compared with WT (eLPS) group.
(TIF)

Figure S2 **Heat inactivation of** ***B. tropicalis*** **allergens has not effect for the allergic airway disease phenotype.** C57BL/6 (WT) mice were sensitized twice with heat-inactivated *B. tropicalis* extract (iBt) or iBt plus eLPS co-adsorbed to alum and challenged twice with Bt. Control group consisted of non-manipulated animals. The experiments were performed 24 h after the last Bt challenge. Enzyme activation assay of heat inactivated extract by hydrolysis of Z-F-R-MCA (**A**); Total bronchoalveolar lavage (BAL) leukocyte counts (**B**); Eosinophils and neutrophils counts in the BAL fluid (**C**); Total IgE in serum (**D**); Results are expressed as mean ±SEM for groups of five mice and are representative of two experiments. *Significant difference (*p*<0.05) when compared with the control group.
(TIF)
