# Supplementary figures and images for: Endotoxin Exposure during Sensitization to Blomia tropicalis Allergens Shifts TH2 Immunity Towards a TH17-Mediated Airway Neutrophilic Inflammation: Role of TLR4 and TLR2
Source: PLoS One. 2013 Jun 21;8(6):e67115. doi: 10.1371/journal.pone.0067115 (PMC3689683; doi:10.1371/journal.pone.0067115)

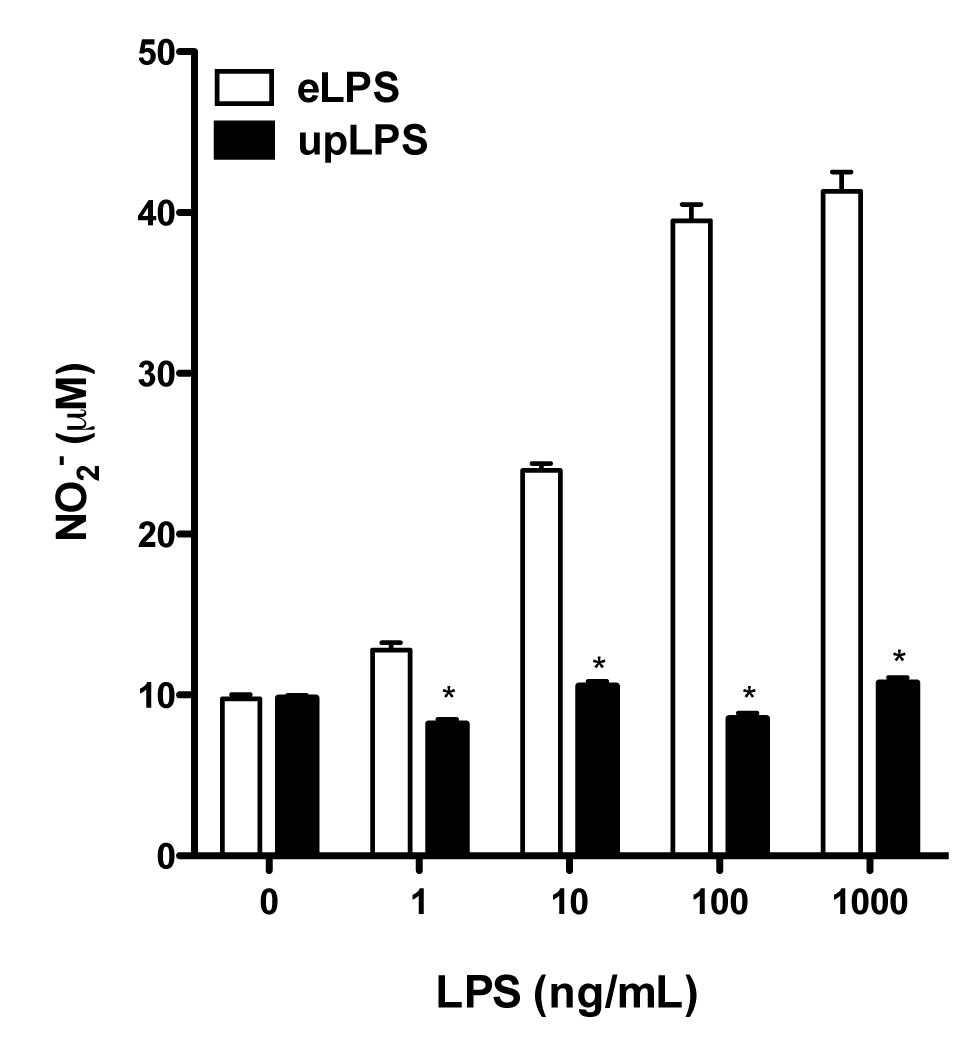

Supplement: Figure S1 — Endotoxin LPS (eLPS) and Ultra-pure LPS (upLPS) have distinct effects on in vitro activation of TLR4−/− macrophages. Thioglycollate-elicited peritoneal macrophages from C57BL/6 (WT) or TLR4−/− mice were incubated with medium only or stimulated in vitro with IFN-γ alone (10 ng/mL) or in the presence of different concentrations of eLPS or upLPS. NO2 − production was evaluated after 48 h culture by Griess reaction. *Significant difference (p<0.05) when compared with WT (eLPS) group. (TIF) [file pone.0067115.s001.tif]

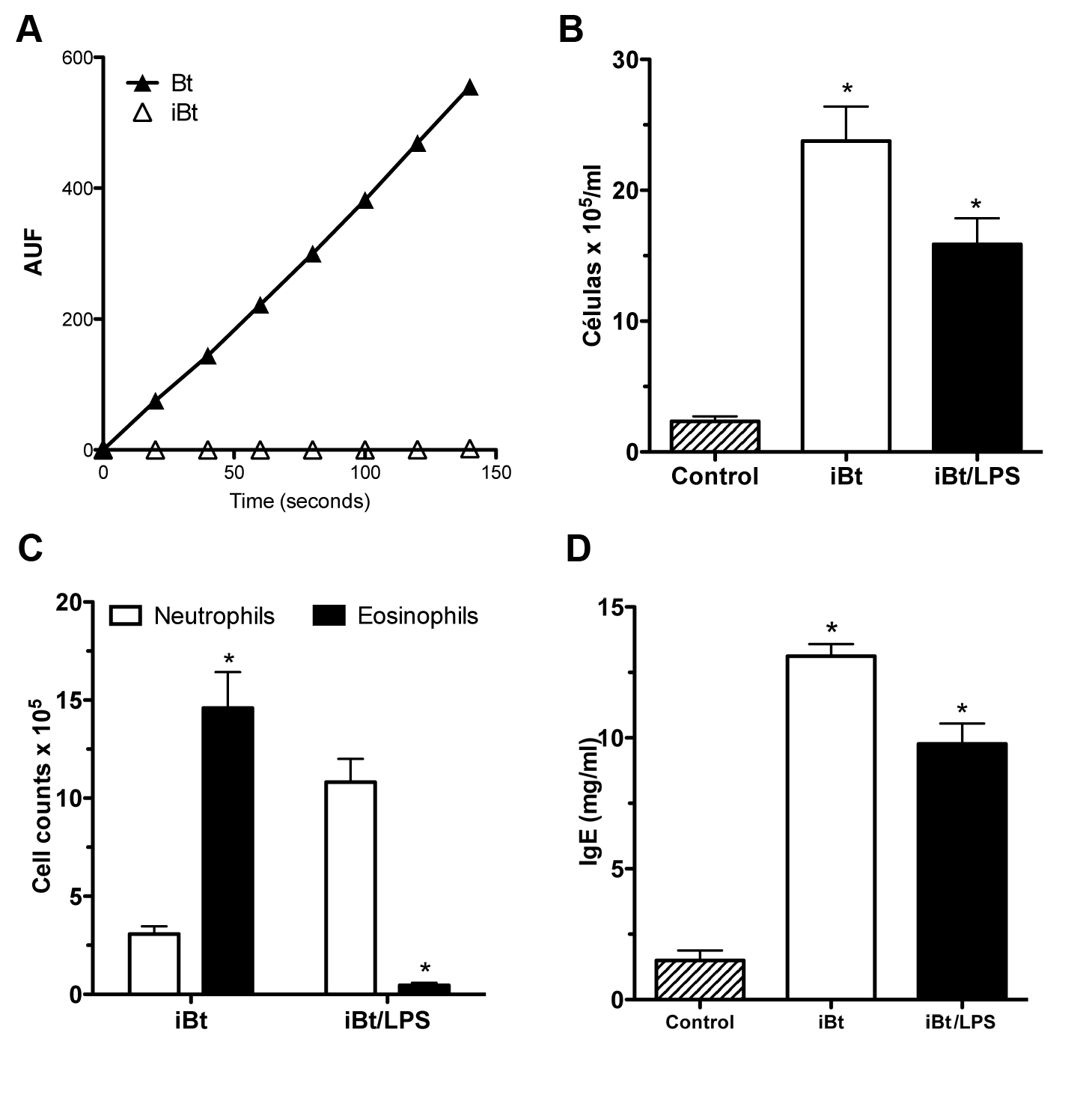

Supplement: Figure S2 — Heat inactivation of B. tropicalis allergens has not effect for the allergic airway disease phenotype. C57BL/6 (WT) mice were sensitized twice with heat-inactivated B. tropicalis extract (iBt) or iBt plus eLPS co-adsorbed to alum and challenged twice with Bt. Control group consisted of non-manipulated animals. The experiments were performed 24 h after the last Bt challenge. Enzyme activation assay of heat inactivated extract by hydrolysis of Z-F-R-MCA (A); Total bronchoalveolar lavage (BAL) leukocyte counts (B); Eosinophils and neutrophils counts in the BAL fluid (C); Total IgE in serum (D); Results are expressed as mean ±SEM for groups of five mice and are representative of two experiments. *Significant difference (p<0.05) when compared with the control group. (TIF) [file pone.0067115.s002.tif]
